# Action Mechanism of Inhibin α-Subunit on the Development of Sertoli Cells and First Wave of Spermatogenesis in Mice

**DOI:** 10.1371/journal.pone.0025585

**Published:** 2011-10-05

**Authors:** Kailai Cai, Guohua Hua, Sibtain Ahmad, Aaixin Liang, Li Han, Canjie Wu, Feifei Yang, Liguo Yang

**Affiliations:** 1 Key Laboratory of Agricultural Animal Genetics, Breeding and Reproduction, Education Ministry of China, Huazhong Agricultural University, Wuhan, People's Republic of China; 2 Department of Livestock Management, University of Agriculture, Faisalabad, Pakistan; University of South Florida College of Medicine, United States of America

## Abstract

Inhibin is an important marker of Sertoli cell (SC) activity in animals with impaired spermatogenesis. However, the precise relationship between inhibin and SC activity is unknown. To investigate this relationship, we partially silenced both the transcription and translation of the gene for the α-subunit of inhibin, *Inha*, using recombinant pshRNA vectors developed with RNAi-Ready pSIREN-RetroQ-ZsGreen Vector (Clontech Laboratories, Mountain View, Calif). We found that *Inha* silencing suppresses the cell-cycle regulators Cyclin D1 and Cyclin E and up-regulates the cell-cycle inhibitor P21 (as detected by Western blot analysis), thereby increasing the number of SCs in the G1 phase of the cell cycle and decreasing the amount in the S-phase of the cell cycle (as detected by flow cytometry). *Inha* silencing also suppressed *Pdgfa*, *Igf1*, and *Kitl* mRNA levels and up-regulated *Tgfbrs*, *Inhba*, *Inhbb*, *Cyp11a1*, *Dhh*, and *Tjp1* mRNA levels (as indicated by real-time polymerase chain reaction [PCR] analysis). These findings indicate that *Inha* has the potential to influence the availability of the ligand inhibin and its antagonist activin in the SC in an autocrine manner and inhibit the progression of SC from G1 to S. It may also participate in the development of the blood–testis barrier, Leydig cells, and spermatogenesis through its effect on *Dhh*, *Tjp1*, *Kitl*, and *Pdgfa*. Real-time PCR and Western blot analyses of *Inha*, *Inhba*, and *Inhbb* mRNA and Inha levels over time show that *Inha* plays an important role in the formation of round spermatid during the first wave of spermatogenesis in mice.

## Introduction

The Sertoli cell (SC) acts as the central regulator of testicular development and function. SCs are the only cells in the fetal gonad to undergo differentiation. This event facilitates the formation of the seminiferous tubules and prevents germ cells from entering meiosis to undergo differentiation into Leydig cells [1]. SCs also regulate the proliferation and development of primordial germ cells during the fetal period [2]. Clearly, the regulation of SC proliferation and activity during development and in the adult animal is crucial for normal adult fertility [3]. Therefore, the mechanisms underlying SC development warrant further investigation.

In the male, inhibin is produced mainly by SCs [4]–[6]. This protein acts in an endocrine manner to negatively regulate the synthesis and release of follicle-stimulating hormone (FSH) from the anterior pituitary gland [7]–[10]. It has been shown that FSH can maintain spermatogenesis in hypophysectomized rats [11] and is one of the main hormones to stimulate spermatogenesis [12]. Inhibin is a heterodimer containing a unique α-subunit (Inha) that serves as its functional component. An Inha disulphide is linked to one of 2 β subunits (βA and βB) of inhibin to form inhibin A or inhibin B, respectively [9], [13]. The expression and secretion of inhibin B correlate with SC activity, sperm number, and spermatogenic status and are inversely correlated with FSH. [14]. Moreover, inhibin B and FSH can serve as markers of SC activity in animals with impaired spermatogenesis [15]. The extent of SC proliferation in the fetus and in the juvenile testis is a primary determinant of adult spermatogenesis. However, the precise relationship between inhibin and SC development and between inhibin and spermatogenesis-related genes and testis development has not been studied.

To date, inhibin immunization has been shown to increase total sperm production in rabbits [16], bulls [17]–[19], and pigs [20]. Recently, RNA interference (RNAi)–which has served as a powerful tool for exploring gene expression and identifying protein activity in a wide range of gene knockout models in mammals [21]–[25]—has also been used to suppress *inhibin* expression, thereby improving total sperm production, and to study inhibin activity.

The objective of this study was to determine the role of inhibin in the regulation of mouse SC development, the expression of spermatogenesis-related genes, and the expression of *Inha* mRNA and protein production when sperm first appear in the mouse testis. Our goal is to provide a foundation for studying the mechanisms of SC development and spermatogenesis in order to improve current methods of sperm production.

## Results

### Identification and validation of *Inha* RNAi recombinant plasmids in SC cell culture

**Figure 1:**
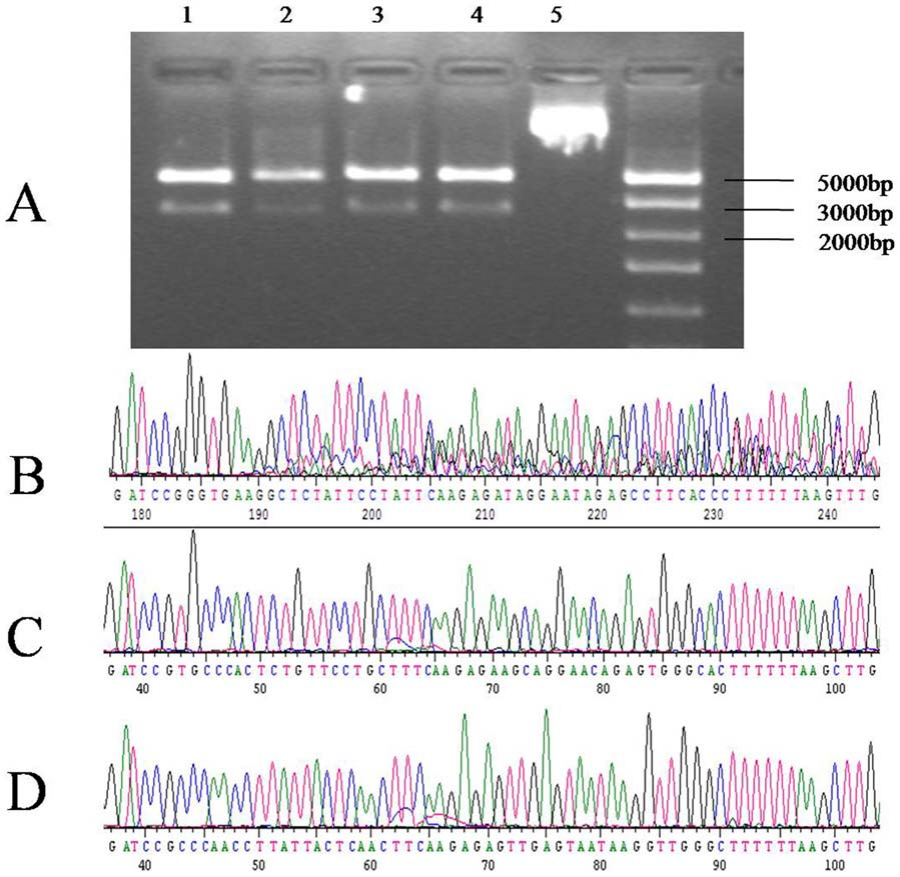
Restriction mapping and sequencing analysis. Restriction mapping analysis shows (**A**) the pshRNA-1 (Lane 1), pshRNA-2 (Lane 2), pshRNA-3 (Lane 3), and pshRNA-negative (Lane 4), RNAi-Ready pSIREN-RetroQ-ZsGreen Vector (Lane 5) plasmids and sequencing of plasmids pshRNA-1 (**B**), pshRNA-2 (**C**), and pshRNA-3 (**D**).

**Figure 2:**
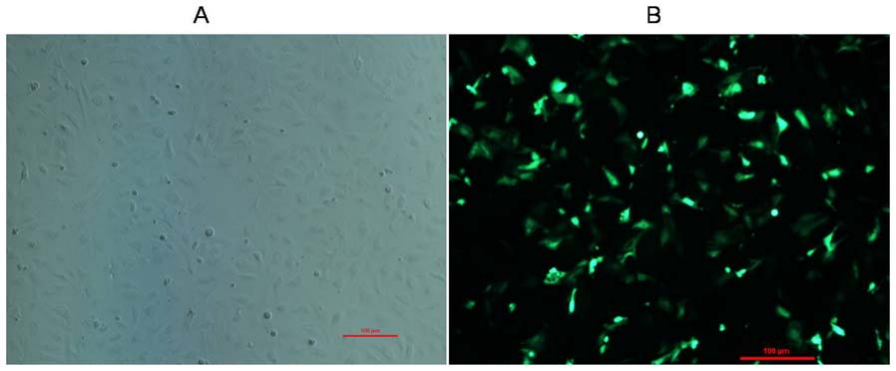
RNAi recombinant plasmids expressed in SCs. GFP expression in SCs with (**B**) or without (**A**) transfection of RNAi recombinant plasmids 48 h after transfection. The scale bar represents 500 µm.

**Figure 3:**
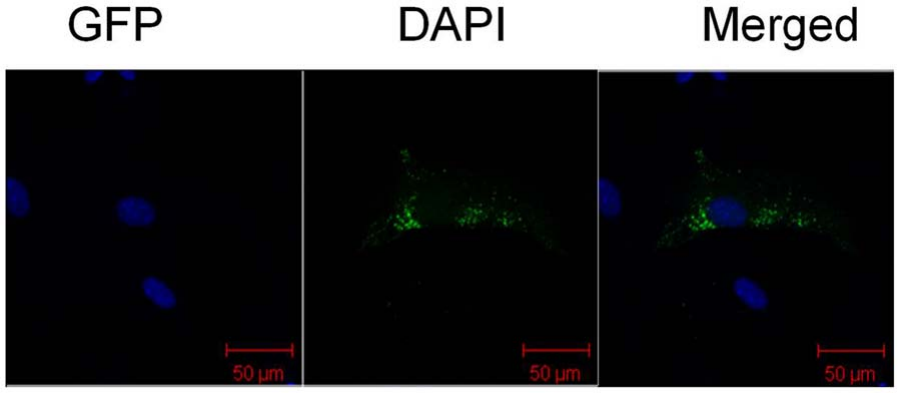
GFP localization in SCs. RNAi recombinant plasmids in SCs transfected using the Lipofectamine™2000 Kit (Invitrogen; Carlsbad, Calif) and stained with DAPI 48 h after transfection. Images were taken using a confocal microscope. GFP and DAPI fluorescence using different filters and the merged pictures are shown separately. Green fluorescence appears only in the cytoplasm; blue fluorescence is seen in the nuclei. The scale bar represents 50 µm.

Three Inha RNAi recombinant plasmids were identified by restriction analysis and sequencing. There is a HindIII site at position 2456 in the pSIREN-RetroQ - ZsGreen plasmid, and a HindIII site inserted in the hairpin fragment of the shRNA and MluI site in the pshRNA- negative. Analysis of 2 fragments (2500 and 4100 base pairs, respectively) released from the recombinant plasmids through digestion with homologous restriction enzymes revealed that the siRNAs were inserted correctly (A, Fig. 1); these clones were further confirmed by sequencing. No mutations were found in the 3 hairpin fragments (Fig. 1B–D). SCs were transfected with this vector, which expresses a *Zoanthus* spp. green fluorescent protein engineered for brighter fluorescence (maximum excitation: 496 nm; maximum emission: 506 nm) [26]. SCs transfected with the *Inha* RNAi recombinant plasmids (pshRNA-1, pshRNA-2, pshRNA-3, and pshRNA-negative) had a brighter green fluorescence 12 h, 24 h, and 48 h after transfection, the fluorescence being brightest 48 h after transfection (Fig. 2). In SCs transfected with the RNAi recombinant plasmids, green fluorescence was observed only in the cytoplasm; blue fluorescence was observed in the nuclei, reflecting the DAPI stain (A–C, Fig. 3). The presence of GFP indicated the transfection had worked and that GFP was expressed and localized to the cytoplasm. These observations indicate that the Inha RNAi recombinant plasmids were expressed normally in the SCs.

### Identification of the effect of *Inha* RNAi recombinant plasmids

**Figure 4:**
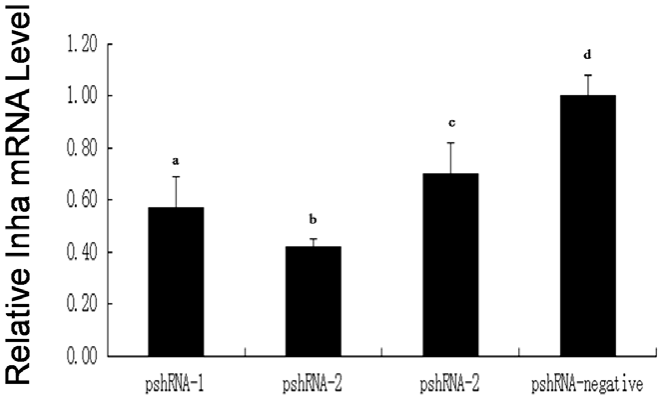
*Inha* mRNA expression in transfected cells. The *Inha* mRNA levels in SCs transfected with RNAi recombinant plasmids pshRNA-1, pshRNA-2, pshRNA-3, or pshRNA-negative 48 h after transfection. Data are presented as the mean ± SEM (n = 3 in each group). For bars with different letters (a, d; b, d; c, d), the difference was significant (*P*<0.01).

**Figure 5:**
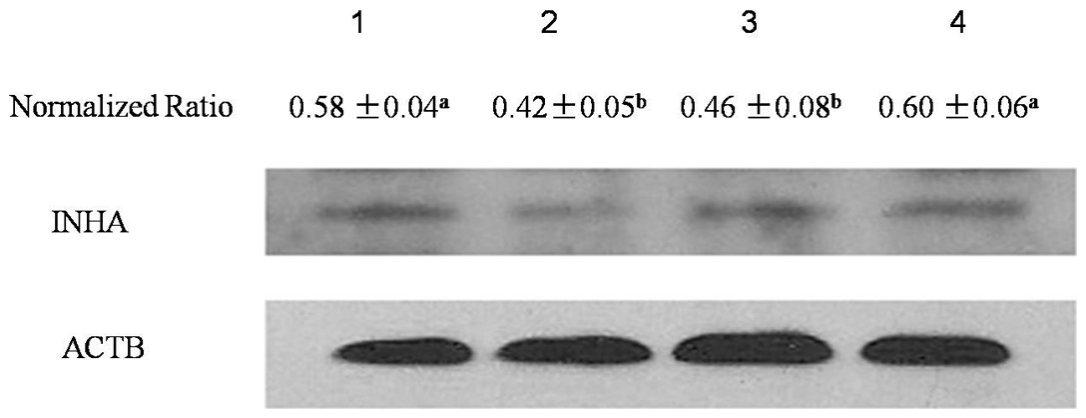
INHA protein levels in SCs. Inha levels in SCs transfected with plasmids pshRNA-1, pshRNA-2, pshRNA-3, and pshRNA-negative 48 h after transfection. Lanes 1 to 4 represent the pshRNA-1, pshRNA-2, pshRNA-3, and pshRNA-negative plasmids, respectively. The normalized ratio for INHA was calculated by dividing the mean signal intensity for 3 biological replicates by the mean signal intensity with ACTB. Data are presented as the mean ± SEM, with different letters (a, b) was significant (*P*<0.05), as evaluated using Student's paired t test.

*Inha* mRNA was down-regulated in the presence of the plasmids pshRNA-1, pshRNA-2, and pshRNA-3 (Fig. 4), with pshRNA-2 having the greatest effect. The silencing efficiency of these plasmids was 43%, 58%, and 30%, respectively, compared with the pshRNA-negative plasmid. Western blotting was performed to investigate *Inha* protein levels (Fig. 5). These results indicated that *Inha* protein was inhibited mostly by pshRNA-2 compared with the pshRNA-negative. These data agree with the mRNA expression changes shown by quantitative real-time PCR analysis and suggested that the inhibitory effects occurred at the post-transcription level. To determine the effect of pshRNA-2 on inhibin secretion by SCs, we examined SC media collected 48 h after being transfected with pshRNA-2 or pshRNA-negative plasmids. Inhibin B concentrations decreased significantly in media transfected with pshRNA-2 compared with those transfected with the pshRNA-negative plasmid (25.92±3.64 pg/mL vs 33.31±4.86 pg/mL; *P* = 0.013), indicating that pshRNA-2 could significantly reduce the amount of inhibin B secreted by SCs in *vitro*.

### *Inha*-regulated SC cell-cycle progression in cultured SCs

To determine whether *Inha* regulates growth-factor–induced cell-cycle progression, we stained SCs with propidium iodide/RNase A then carried out fluorescence-activated cell sorting (FACS). In the presence of pshRNA-2 compared with the pshRNA-negative plasmid, the number of S-phase SCs decreased significantly (13.89±0.08 vs 17.04±0.41; *P* = 0.008), the number of G1-phase SCs increased significantly (62.58±0.04 vs 60.50±0.39; *P* = 0.02) (Table 1), and the proportion of cells in the S and G2M phases of the cell cycle (the proliferative index) decreased significantly (0.37±0.007 vs 0.40±0.07; *P* = 0.023). These findings suggest that Inha regulates the progression in SC through the cell cycle and, thus, can affect their development.

**Table 1:** Effects of *Inha* silencing on the cell cycle in SCs.*

Plasmid Used in Transfection	Cell-Cycle Phase		
	G1	S	G2
pshRNA-2	62.58±0.07**	13.89±0.13**	23.52±0.18
pshRNA-negative	60.50±0.67	17.04±0.7	22.67±0.98

*Each experiment was repeated 3 times. Values represent the mean ± SEM (n = 3 in each group).

**P<0.01, as evaluated by Student's paired t test.

### Effect of cell-cycle regulation after *Inha* silencing

**Figure 6:**
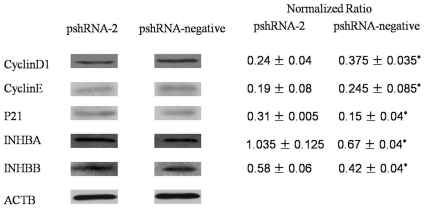
Protein levels in transfected SCs. INHBA, INHBB, Cyclin D1, Cyclin E, and P21 levels in SCs transfected with the pshRNA-2 and pshRNA-negative plasmids 48 h after transfection. The normalized ratio for each protein was calculated by dividing the mean signal intensity from 3 biological replicates by the mean signal intensity with ACTB. Data are presented as the mean ± SEM. *P<0.05, as evaluated using Student's paired t test.

To determine whether *Inha* silencing affects the progression of SCs through the cell cycle, we used Western blot analysis to measure Cyclin D1, Cyclin E, and P21 levels throughout the cell-cycle in SCs exposed to the pshRNA-2 and pshRNA-negative plasmids. We found that Cyclin D1 (*P* = 0.012) and Cyclin E (*P* = 0.029) expression was significant lower and P21 (*P* = 0.030) expression was significant higher in cells transfected with pshRNA-2 compared with the pshRNA-negative plasmid (Fig. 6). Meanwhile, INHBA (*P* = 0.032) and INHBB (*P* = 0.038) expression was significant higher in cells transfected with pshRNA-2 compared with the pshRNA-negative plasmid.

### Effect of *Inha* regulation on gene expression in SCs

**Figure 7:**
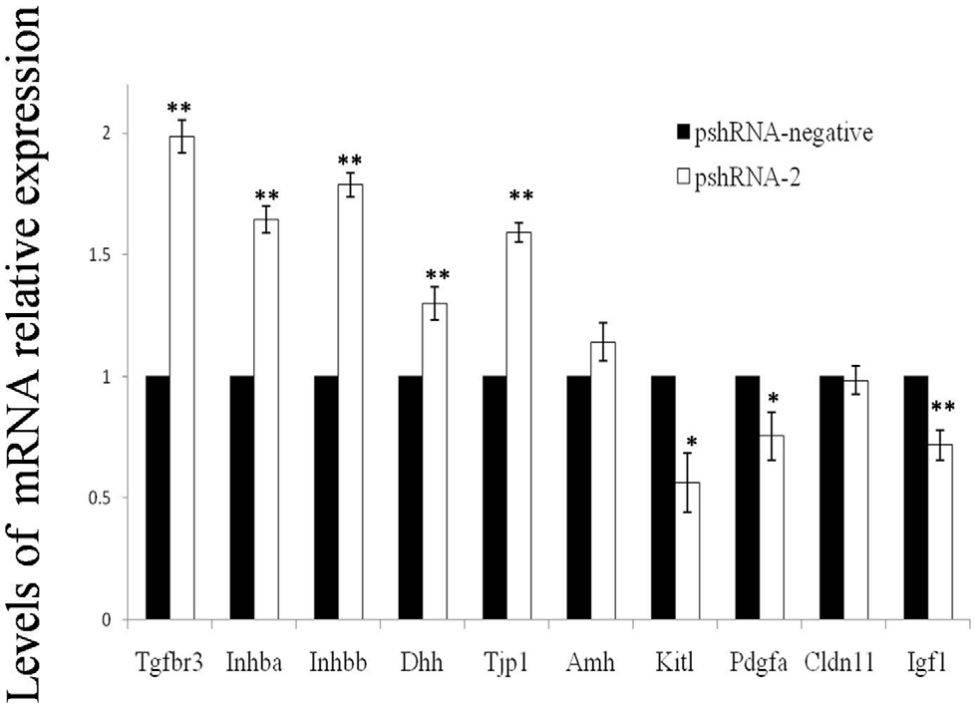
mRNA expression in transfected SCs. Expression of mRNA for *Tgfbr3*, *Inhba*, *Inhbb*, *Dhh*, *Tjp1*, *Kitl*, and *Pdgfa* in SCs 48 h after transfection with the pshRNA-2 and pshRNA-negative plasmids. Data are presented as the mean ± SEM (n = 3 in each group). **P<0.01; *P<0.05, as evaluated using Student's t test.

To determine whether *Inha* silencing affects the expression of spermatogenesis-related genes (which is limited or absent in all cell types except SCs) in the mouse testis and components of the transforming growth factor (TGF)-β superfamily and other cell-cycle factors, we quantified the expression of *Tgfbr3*, *Inhba*, *Inhbb*, *Igf1*, *Dhh*, *Pdgfa*, *Cldn11*, *Kitl*, *Amh* and *Tjp1* mRNA using real-time PCR in cells exposed to pshRNA-2 and pshRNA-negative plasmids. Exposure to the pshRNA-2 versus pshRNA-negative plasmid resulted in significant up-regulation of *Tgfbr3* (*P* = 0 .00012), *Inhba* (*P* = 0 .00029), *Inhbb* (*P* = 0 .00008), *Dhh* (*P* = 0 .01240), and *Tjp1* (*P* = 0 .0001) and a significant down-regulation of *Pdgfa* (*P* = 0.03429), *Igf1* (*P* = 0.00515), and *Kitl* (*P* = 0.01131) (Fig. 7). The change in *Cldn11* and *Amh* expression in the presence of these 2 plasmids was not significant.

### Developmental expression of transcript of *Inha*, *Inhba* and *Inhbb* in postnatal testes

To investigate *Inha*, *Inhba*, and *Inhbb* expression profiles, we carried out a real-time PCR analysis of their mRNA levels in the mouse testis at 1, 7, 10, 14, 21, 28, 35, and 56 days postpartum. We found that *Inha* expression decreased between day 1 and day 10 (*P*<0.05), increased between day 10 and day 21 (*P*<0.05), and then decreased between day 21 and day 28 (*P*<0.05) (Table 2). There was no significant change in expression between day 28 and day 56. *Inha* expression on day 1 was significantly higher than on days 7, 10, 14, 21, 35, or 56 (*P*<0.05, each). *Inhba* and *Inhbb* expression was significantly reduced compared with *Inha* expression on each study day except day 28.

**Table 2:** Relative mRNA expression level of Inha, Inhba and Inhbb.

Gene		Inha	Inhba	Inhbb
Relative mRNA expression level	day1	7.890±2.621Aa	1.893±0.475Ab	0.137±0.067BDb
	day7	2.140±0.257Ba	0.337±0.026Bb	0.180±0.042Bb
	day10	1.300±0.120Ca	0.090±0.010Bb	0.405±0.155Ab
	day14	1.785±0.255BCa	0.213±0.034Bb	0.073±0.017BDb
	day21	3.230±0.356Da	0.230±0.027Bb	0.045±0.009CDb
	day28	0.130±0.028EFb	0.130±0.029Bb	0.023±0.003CDa
	day35	0.640±0.021Fa	0.013±0.003Bb	0.160±0.025BDc
	day56	0.537±0.145Fa	0.016±0.003Bb	0.050±0.011BDc

Relative mRNA expression level of Inha, Inhba and Inhbb from male neonates mice at days 1, 7, 10, 14, 21, 28, 35, and 56 of age.

Values presented by mean ± S.E.M, n = 4 in each age group. In the horizontal row, significance level was p<0.05 between values scripted with different little letters, and not significant with same little letters. In the vertical row, significance level was p<0.05 between values scripted with different big letters, and not significant with same big letters.

### Developmental expression of INHA protein in postnatal testes

**Figure 8:**
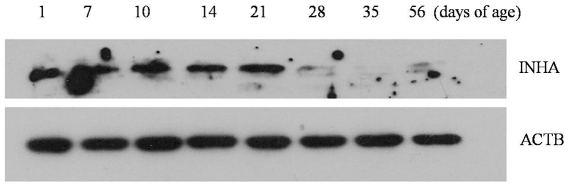
INHA profiles. INHA in male neonate mice on days 1, 7, 10, 14, 21, 28, 35, and 56 post partum. The normalized ratio for INHA protein was calculated by dividing the mean signal intensity from 3 biological replicates by the mean signal intensity with ACTB.

We investigated INHA protein expression profiles using a Western blot analysis of INHA in the testes 1, 7, 10, 14, 21, 28, 35, and 56 days postpartum. We found that INHA levels were higher on days 1, 7, 10, 14, and 21 compared with days 28, 35, and 56 and that expression of this protein was extremely limited after day 28 (Fig. 8).

## Discussion

In the present study, we designed and constructed 3 *Inha* RNAi vectors and stably transfected them into mouse SCs, where they were expressed normally in the cells. *Inha* mRNA and protein expression were significantly inhibited in mouse SCs. The pshRNA-2 plasmid was the most effective in silencing *Inha* mRNA and protein expression. It also significantly decreased inhibin B secretion by cultured SCs. These results indicate that inhibin is partially inhibited by *Inha* silencing. The effectiveness of this plasmid in cultured mice SCs points to a potential in vitro approach to studying the mechanism by which inhibin regulates SC development in polytocous animal cells. It may also serve as a foundation for developing an alternative to inhibin immunization as a means of improving the total sperm count in animals.

Spermatogenesis begins with the differentiation of germ cells into spermatogonia (day 7), which is followed by the production of the early spermatocyte, the late spermatocyte, round spermatid (day 21), the elongated spermatid (day 28), and, finally, the complete sperm (day 35) [27]. Previous studies suggest that this first wave of spermatogenesis is significantly different from later waves [28]–[30]. Inhibin, activin, follistatin and FSH serum levels and testicular production are highly modulated during the first spermatogenic wave in mice [31]. TGF-β superfamily signaling is an integral part of normal testicular development and regulation of the processes leading to the production of fertile sperm [32]. The ligands activin and inhibin belong to the TGF-β superfamily [33], with inhibin acting as a competitive antagonist to activin [34]. During the first wave of spermatogenesis, *Inha* expression was significantly higher than *Inhba* and *Inhbb* expression from day 1 to day 21; the expression of all 3 genes and the Inha protein was very low from day 28 to day 35. At day 56, when the mouse reaches sexual maturity, expression of the 3 genes remained low. This is a new evidence to support that inhibin is very important during this first wave of spermatogenesis, especially before the round spermatid is formed because of the rate of *Inha* expression during the first stage of spermatogenesis.

Our findings may serve as new evidence of the autoregulatory properties of the Inha gene. First the expression of mRNA and protein for both *Inhba* and *Inhbb*, which produce 2 subunits found in both inhibins and activinsincreased significantly after *Inha* silencing was achieved in cultured SCs. Furthermore, *Inha* silencing resulted in a decrease in inhibin levels and possibly an increase in activin levels in cultured mouse SCs. Collectively, these findings demonstrate a novel mechanism for autoregulation of the inhibin-alpha subunit. Evidence for this mechanism is supported by an earlier report that gonadal inhibin can down-regulate the expression of *Inha* in the adrenal gland [35]. Thus, we can speculate that *Inha* has the potential to influence inhibin and activin in a specific autocrine manner in SCs.

SC proliferation begins during the fetal period and declines rapidly during the neonatal period, essentially ending by approximately 16 days postpartum in the mouse [36]. In mammals, the number of SCs that have been established during the prepubertal period determines the final testicular size and the number of sperm that will be produced when the animal reaches sexual maturity [37], [38]. We confirmed that *Inha* silencing is followed by a significant decrease in the number of SCs in the S phase and a significant increase in the number of cells in the G1 phase, as well as a significant decrease in the cell proliferative index. This result indicated that Inha were involved in cell cycle regulaton during G1 to S phase transition. Meanwhile, *Inha* silencing also results in a decrease in Cyclin D1 and Cyclin E and an increase in the cell-cycle inhibitor P21. This result also indicated that Inha had effective on cell cycle regulation protein at G1 to S phase transition. This result was consistent to the flow cytometry. Furthermore, It also reduces the rate of expression of *Igf1*, which has been reported to promote the progression of cells from phase G1 to S [39]. Cyclin-dependent kinases have been proven to be universal regulators of the cell cycle in all eukaryotes. Cyclin D1 is a regulatory subunit of the cyclin-dependent kinases CDK4 and CDK6 and is required for cells to progress from G0/G1 to S [40]; Cyclin E is essential for the G1 to Sphase transition [41]. P21 is a potential inhibitor of G1 cyclin-dependent kinases [42]. We believe that *Inha* has the potential to affect SC development by regulating their progression from G1 to S and to indirectly influence testis development and spermatogenesis.

*Dhh*, *Tjp1*, *Kitl*, *Pdgfa*, *Cldn11* and *Amh* are expressed in SCs but have shown little or no expression in any other cell type in the testis [43]–[48]. Therefore, we can speculate that these genes have important roles in SC and testis development. In previous studies, investigators reported their observation of *Dhh* expression in fetal SCs and their precursors and expression of the gene for its receptor, Ptc, in fetal mouse Leydig cells and myoid cells [43], [49]. A mutation in the Dhh gene may be responsible for the pseudo-hermaphrodite phenotypes of the mutant rat and is probably essential for the development of Leydig cells [50]. The Pdgf family, which consists of 4 ligands (A, B, C, and D) and 2 distinct receptors, appears to affect the differentiation of Leydig cells. One member of this family, Pdgfa, is expressed in both XX and XY gonads 11.5 days post coitus (dpc); by 12.5 dpc, it is strongly expressed in SCs in the seminiferous tubules, whereas its expression in the XX gonad is diminished [51]. Zonula occludens 1, the product of *Tjp1* translation, has been described as a component of the SC barrier or associated with ectoplasmic specialization. It is found mainly in 3 classes of tight junction integral membrane proteins: the occludins, claudins, and junctional adhesion molecules [52]. The tight junctions between SC cells comprise the blood–testis barrier (BTB), which restricts the movement of water, solutes, and immune cells from the circulation into the seminiferous tubules, thereby creating an immunologically unique microenvironment for spermatogenesis [53]. Stem cell factor (SCF), also known as the kit ligand (the product of *Kitl*), is the ligand of c-kit. The SCF/c-kit system is involved in the development of the testes and regulation of spermatogenesis; thus, it is an important survival factor [54]. In this study, we demonstrated that *Inha* silencing can significantly up-regulate *Dhh* and *Tjp1* mRNA and down-regulate *Pdgfa* and *Kitl* mRNA. Thus, Inha may participate in the construction of the blood–testis barrier, in the development of Leydig cells, and in spermatogenesis. Furthermore, its activity may correlate with that of Dhh, Tjp1, Kitl, and Pdgfa. Additional research is necessary to understand the mechanism of action underlying the changes in gene expression associated with Inha silencing.

In conclusion, our analysis of the temporal expression of *Inha*, *Inhba*, and *Inhbb* mRNA and the Inha protein has revealed that Inha is important for the development of the round spermatid during the first wave of spermatogenesis in the mouse. Using *Inha* RNAi recombinant plasmid-transfected SCs, we found that Inha has the potential to influence SC inhibin and activin levels in a specific autocrine manner and affect SC development by regulating their progression from G1 to S. We also found that *Inha* silencing significantly affects the expression of *Dhh*, *Tjp1*, *Kitl*, and *Pdgfa*, which are expressed in SCs but have shown little or no expression in any other cell types in the testis and are involved in the construction of the blood–testis barrier, in Leydig cell development, and spermatogenesis. Additional studies will be required to determine whether this process can serve as a basis for studying the role of inhibin in spermatogenesis and finding an alternative to inhibin immunization to enhance sperm production.

## Materials and Methods

### Experimental animals

Male specific pathogen-free Kunming mice were procured from the Centre of Laboratory Animals of Hubei Province (Wuhan, PR China). This study was approved by the Ethical Committee of the Hubei Research Center of Experimental Animals (Approval ID: SCXK(Hubei)2008-0005). In this study, animals were treated in accordance with the NIH Guide for the Care and Use of Laboratory Animals.

### Isolation and culture of primary Sertoli cells (SCs)

Briefly, testes were aseptically removed and placed in Petri dishes containing Hank's balanced salt solution (HBSS) , removed the tunica albuginea from the testes, cut the testes into small pieces and transfer the seminiferous tubules to a new 60-mm Petri dishes containing 4–5 ml of 2 mg/ml collagenaseIV/DNase solution (Sigma, USA), and incubated at 37°C in a CO_2_ incubator until the tubules separated (about 20 min), and then washed with HBSS The tissues were resuspended in calcium-and magnesium-free HBSS and further digested with 0.25% trypsin-0.02% EDTA (1∶1) for 20 to 30 min at 37°C. Following digestion, the mixture was passed through a 200 µm stainless mesh, and then washed with HBSS. The enzyme solution was decanted by centrifugation at 200 *g* for 10 min. The cell pellet obtained was resuspended in DMEM with 15% FBS and allowed to settle. The settled cells were cultured at 37°C in a 5% CO2 atmosphere for three days in DMEM supplemented with 10% FBS (Invitrogen), 100 U/ml penicillin, 100 ug/ml streptomycin.

### Selection of target sequence and Construction of RNA interference vector

The complete coding sequence of mouse inhibin alpha (*Inha*, NM_010564) was retrieved from the NCBI GenBank database. Three siRNA target sites were selected according to the siRNA program [55], [56] at positions 273, 772, and 1237 in the coding region (Table 3), and these three selected sequences were submitted to a BLAST search against the mouse genome to ensure their specificity. To obtain short hairpin RNA, a typical oligonucleotide that has 5 bases containing a restriction site at its 5′ end, 19 bases of sense strand, 7 to 9 bases of hairpin loop, 19 bases of antisense strand, 6 bases of terminator, and 6 bases corresponding to a unique HindIII restriction site (resulting in a total length of 65 bases) and 2 complementary oligonucleotides were synthesized. These were annealed and inserted into the BamHI and EcoRI sites of the RNAi-Ready pSIREN-RetroQ-ZsGreen Vector (BD Biosciences, Clontech, Mountain View, CA). The recombinant plasmids were designated as pshRNA-1, pshRNA-2, and pshRNA-3, respectively. A plasmid (pshRNA-negative) encoding a hairpin siRNA comprising of sequence without sense that has not been found in the mouse or human genomes was used as the negative control.

**Table 3:** Target sequences of mouse inhibin alpha (*Inha*).

Name	Target sequence (5′→3′)	Position on cds
siRNA-1	GGTGAAGGCTCTATTCCTA	273
siRNA-2	TGCCCACTCTGTTCCTGCT	772
siRNA-3	CCCAACCTTATTACTCAAC	1237
Negative control	TGGACATAGGCGACGTGT	

### Transfection of Recombinant pSIREN Vectors

Plasmids pshRNA-1, pshRNA-2, pshRNA-3 and pshRNA-negative in a super-coiled form were obtained using EndoFree Plasmid Kit (Tiangen, Beijing), respectively. One day before transfection, 0.5–2×10^5^ Sertoli cells were seeded in 500 µl of growth medium without antibiotics in 12-well plate such that cells would be 90–95% confluent at the time of transfection. Four groups of Sertoli cells were observed in total to transfect designated on the basis of plasmid names: pshRNA-1, pshRNA-2, pshRNA-3 and pshRNA-negative, respectively. Transfection was performed using Lipofectamine™ 2000 Kit (Invitrogen) according to manufacturer's instructions. After 8 h, transfection medium was changed into growth medium without antibiotics. To examine transfection efficiency, cells transfected with vectors were observed under a fluorescent microscope to detect the expression of GFP. Cells were collected for RNA extraction, protein extraction and other experiments. The medium was collected at 48 h after transfection, and stored at −20°C for hormone analysis.

### DAPI staining for transfected cells

DAPI (4′ 6-diamidino-2-phenylindole, Sigma) was used as a DNA-specific probe, which passed through the cell membrane. SCs grown on glass coverslips in 6-well culture plate at 48 h after transfection were washed twice with PBS, and fixed in methanol for 5 min at room temperature. Cells were washed three times with PBS for 10 min, and then covered with 1 mg/ml of DAPI. After staining in a dark chamber for 10 min, the DAPI solution was removed by rinsing with PBS. Slides were finally analyzed using a Confocal laser scanning microscope (LSM 510 Meta instrument, Zeiss).

### Hormonal Analysis

The medium collected from pshRNA-2 and pshRNA-negaive at 48 after transfection was analysed for inhibin B (INB) concentration using GBD® INB ELISA kit (Groundwork Biotechnology Diagnosticate Ltd, USA). The concentration of INB in the samples was determined by comparing the O.D. of the samples to the standard curve.

### RNA extraction and real-time PCR

Total cellular RNA was extracted from collected cells (transfected with pshRNA-1, pshRNA-2, pshRNA-3 and pshRNA-negative at 48 h after culture) using RNAprep pure Cell Kit (Tiangen, Beijing). Testes tissue total RNA was extracted using RNAprep pure Tissue Kit (Tiangen, Beijing) from male neonates at days 1, 7, 10, 14, 21, 28, 35, and 56 of age. At each age group, four mice from different breeding pairs were used to obtain the testes. The first-strand cDNA was synthesized by using first strand cDNA synthesis kit (code NO. FSK-100; Toyobo Co.). Quantitative real-time PCR was carried out using SYBR Green (SYBR Green Realtime PCR Master Mix QPK-201; Toyobo Co.). Specific PCR settings were used in a Bio-Rad iQ5 Real Time PCR system. Melting curve analyses were performed after real-time PCR reactions to monitor PCR product purity.Primer pairs (Table 4) were used for the amplification to analysemRNA relative expression levels. The threshold cycle (CT) numbers were determined for the amplified cDNA for each investigated mRNA and for the house keeping gene, β-actin in each sample during real-time PCR. The relative mRNA expression levels calculated using the formula: 2^−ΔΔ^CT [57].

**Table 4:** Sequences of primer pairs for quantitative real-time PCR.

Gene	Gene bank	Primer sequences (5′–3′)
Inha	NM_010564	Forward CTCGAAGACATGCCGTTGG
		Reverse AGCT GGCTGGTCCTCACAG
β-actin	NM_007393	Forward GGCTGTATTCCCCTCCATCG
		Reverse CCAGTTGGTAACAATGCCATGT
Tgfbr3	NM_011578	Forward GTTCCTGCTCAACTCCCCAC
		Reverse AGCTGGCTGGTCCTCACAG
Inhba	NM_008380	Forward TGAATGAACTCATGGAGCAGACC
		Reverse AGCTGGCTGGTCCTCACAG
Inhbb	NM_008381	Forward CGCGTCTCCGAGATCATCAG
		Reverse AGCTGGCTGGTCCTCACAG
Dhh	NM_007857	Forward TGGCACTCTTGGCACATATC
		Reverse GGCATACTGGGCACAAACT
Kitl	NM_013598	Forward GTAATAGGAAAGCCGCAAA
		Reverse CAAAGCCAATTACAAGCGA
Pdgfa	NM_008808	Forward GACGGTCATTTACGAGATACCTC
		Reverse CTACGCCTTCCTGTCTCCTC
Tjp1	NM_009386	Forward GCCGCTAAGAGCACAGCAA
		Reverse TCCCCACTCTGAAAATGAGGA

### Western Blotting

SCs transfected with the recombinant RNAi vectors were collected at 48 h after transfection, washed in PBS, lyzed in RIPA buffer (Santa Cruz) containing protease inhibitors cocktail (Santa Cruz), incubated for 1 h at 4°C, and centrifuged at 12000 *g* for 10 min, to remove cellular debris, respectively. Testes tissue explants were homogenized in 10 mL of lysis buffer (10% SDS in PBS containing protease inhibitors) and placed on ice for 2 h with vortexing every 10 min. Samples were centrifuged at 12000 *g* for 30 min. Total protein concentrations were determined by BCA-assay (Pierce, Rockford, USA), and 50 µg of total protein was subjected to gel electrophoresis. Proteins were separated on a 12% polyacrylamide gel and transferred to PVDF membranes (Millipore, Bedford, MA). After blocking in PBS supplemented with 5% skimmed milk (Sigma-Aldrich) and 0.05% Tween 20 (Sigma-Aldrich), membranes were incubated overnight at 4°C with primary antibody. Anti-inhibin alpha (1∶300 dilution; Biosynthesis, Beijing), anti-CyclinD1 (1∶300 dilution; Santa Cruz); anti-CyclinE (1∶500 dilution; Boster, Beijing); anti- P21 (1∶500 dilution; Boster, Beijing); anti- INHBA (1∶100 dilution; PTG, Chicago, USA) ; anti- INHBB (1∶200 dilution; PTG, Chicago, USA) Abs was used for primary antibody. After incubation with the primary antibody, membranes were washed three times with PBS containing 0.1% Tween 20, incubated for 1 h at room temperature with 3000-fold diluted HRP labeled goat anti-rabbit secondary antibodies (KPL), and washed three times with PBS containing 0.1% Tween 20. After washing, blots were developed using the ECL Western Blotting detection system (Amersham Biosciences, Piscataway, NJ), and then exposed to X-ray film for visualization of the protein bands. PVDF blots were then stripped of bound antibodies and treated with mouse ACTB antibody (1∶1000 dilution; Santa Cruz) for normalization. The band intensities were measured with AlphaEaseFC software (Alpha Innotech, USA).

### Flow cytometry and cell cycle analysis

SCs transfected with the RNAi vectors were harvested at indicated time intervals, washed with PBS, fixed in ice-cold 75% ethanol overnight at 4°C, washed with PBS and cells stained using propidium iodide/RNase A solution at 37°C in dark for 30 min. Flow cytometry analyses were conducted using a BD FACSCalibur (Becton, Dickinson and Company, USA) and ModFit LT for Mac V3.0 software. For each determination, a minimum of 20,000 cells was analyzed. All experiments were repeated independently at three times.

### Statistical analysis

Data was analyzed using ANOVA, followed by least significant difference t-test (LSD-t) multiple comparisons using SPSS (Version 17.0; SPSS, Chicago, IL, USA). Unless stated otherwise, data are presented as the mean ± SEM. Differences were considered significant at P<0.05.
