# Correction: Action Mechanism of Inhibin α-Subunit on the Development of Sertoli Cells and First Wave of Spermatogenesis in Mice

**DOI:** 10.1371/annotation/2eaf554d-3c4d-429f-a54c-76f31fd5519f

**Published:** 2012-08-08

**Authors:** Kailai Cai, Guohua Hua, Sibtain Ahmad, Aaixin Liang, Li Han, Canjie Wu, Feifei Yang, Liguo Yang

The authors would like to provide some further information regarding the methodology employed and the results reported in the article, and also correct some statements in the Introduction and Discussion. The amendments are outlined below.

Clarifications regarding statements in the Introduction section-

In the second line of the Introduction, the sentences meant to indicate that Sertoli cells are the first cells to differentiate recognizably in the indifferent fetal gonad, an event which enables seminiferous cord formation, prevention of germ-cell entry into meiosis and differentiation and function of the Leydig cells.

In the third sentence of the Introduction, the sentence should be written as "Sertoli cells are the first cells to differentiate recognizably in the indifferent fetal gonad, an event which enables seminiferous cord formation, prevention of germ-cell entry into meiosis and differentiation and function of the Leydig cells (1) ".

From Line 26 of the Introduction, Alpha-inhibin is a tumour-suppressor gene with gonadal specificity in mice from the reference, but in the article we studied the effect of inhibin on SC development and some spermatogenesis related gene expression level by RNAi of Inha. This sentence should be revised as “However, the precise relationship between inhibin and SC development and between inhibin and spermatogenesis-related gene and testis development requires further study”

Clarifications in relation to statements in the Discussion-

We analyzed the mRNA level of Inha, Inhba, and Inhbb from day1 to day 56 but also the relative mRNA level among them by real time PCR and Inha protein level by Western blot. These results were in part similar to those reported in reference 31. Our evidence supports the role of inhibin during the first wave of spermatogenesis, especially before the round spermatid is formed because of the rate of inha expression during the first stage of spermatogenesis.

Clarifications regarding the Methods and Results-

In the "inha- regulated SC cell-cycle progression in cultured SCs’ section of the article, under the Results, the proportion of cells in the S and G2M phases of the cell cycle was calculated by S+G2/G1+G2+S. The data was from Table1 and p=0.023 was calculated by student’s t test.

Under the Results section of the article, the statement "these data agree with the mRNA expression changes shown by quantitative real-time PCR analysis and suggested that the inhibitory effects occurred at the post-transcription level" refers to the fact that’s both the mRNA level and protein levels were reduced, the protein levels reduced belong to post-transcriptional regulation.

In the ‘Figure 2 and the figure legend section of the article, under the results, the presence of GFP indicated the transfection had worked and RNAi recombinant plasmid was expressed normally in the SCs. Fig. 2A showed that cells were not transfected with the RNAi recombinant plasmids. Fig. 2B showed that cells were transfected with the RNAi recombinant plasmids. The figure legend should be corrected as 100um.

We would also like to provide some details regarding the age of the animals used to obtain the Sertoli cells, these were male mice at age 10-12 days. The protocol employed t obtain the cells was the same as that reported in this article: Yin Z, Chen D, Hu F, Ruan Y, Li J, Wang L, Xiang Y, Xie L, Wang X, Ichim TE, Chen S, Chen G （2009）Cotransplantation with xenogenetic neonatal porcine sertoli cells significantly prolongs islet allograft survival in nonimmunosuppressive rats. Transplantation 88:339-345.

In addition, the authors would like to provide some clarification regarding the data reported in Figure 6. The bands for each protein in the two pictures were not generated at the same time and as a result, each of the bands were reported separately, the authors are providing the raw blots from which the figure was generated.

The revised version of Figure 6 in connection with this text can be viewed here: 

**Figure:**
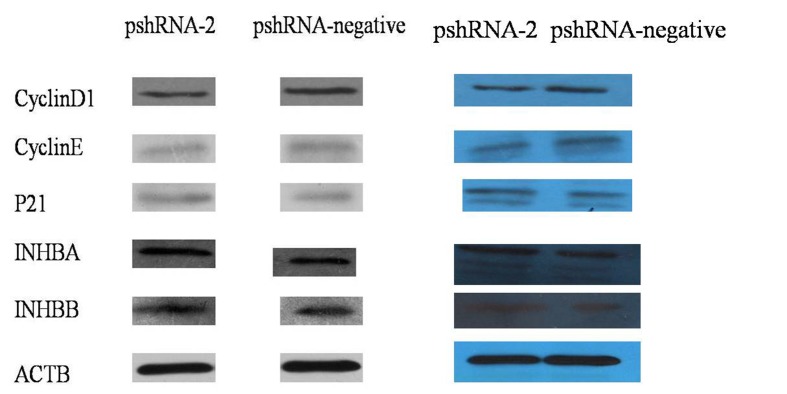


[^] 

